# Cognitive emotion regulation strategies as predictors of short video addiction in children aged 10–14: a descriptive and correlational study

**DOI:** 10.1007/s00431-026-06930-6

**Published:** 2026-04-22

**Authors:** Özge Bicil, Remziye Semerci Şahin, Gülzade Uysal

**Affiliations:** 1 Department of Nursing, Faculty of Health Sciences, Sakarya University of Applied Sciences, Sakarya, Turkey; 2 Department of Pediatric Nursing, School of Nursing, Koç University, İstanbul, Turkey

**Keywords:** Short video addiction, Cognitive emotion regulation, Self-blame, Catastrophizing, Emotion regulation in children

## Abstract

The rapid rise of short video consumption among children and adolescents has raised concerns regarding its associations with cognitive and emotional functioning. This study aimed to examine whether cognitive emotion regulation strategies predict short video addiction levels in children aged 10–14 and to explore the relationship between specific emotion regulation strategies and short video addiction. A descriptive and correlational research design was employed. The study was conducted between January and June 2025 with 202 middle school students aged 10–14. Participants completed an Information Form, the Short Video Addiction Scale (SVAS), and the Turkish Child Version of the Cognitive Emotion Regulation Questionnaire (CERQ-k). Descriptive statistics, correlation analyses, and multiple linear regression analyses were performed. The mean age of the participants was 12.12 years (SD = 1.24). Most were female (52.97%) and enrolled in the 7th grade (30.20%). SVAS scores were positively correlated with self-blame (*r* = .21, *p* < .05), putting into perspective (*r* = .20, *p* < .05), and catastrophizing (*r* = .24, *p* < .01), indicating that higher levels of short video addiction were associated with greater reliance on maladaptive cognitive emotion regulation strategies. Regression analysis identified catastrophizing as a significant predictor of short video addiction scores (*B* = 0.59, *p* < .05), indicating that greater use of catastrophizing was associated with higher levels of short video addiction.

*Conclusion*: The findings indicate that maladaptive cognitive emotion regulation strategies, particularly self-blame and catastrophizing, are associated with higher levels of short video addiction among children. These results highlight the importance of interventions that strengthen adaptive emotion regulation skills as a potential approach to preventing or reducing problematic short video use in childhood.

**Table:** 

**What is Known:** • *Short video use is increasingly common among children and may be associated with cognitive and emotional functioning.* • *Maladaptive cognitive emotion regulation strategies, such as self-blame and catastrophizing, are linked to poorer emotional and behavioral outcomes in children.* **What is New:** • *Maladaptive cognitive emotion regulation strategies are associated with higher levels of short video addiction in children aged 10–14.* • *Catastrophizing significantly predicts short video addiction, identifying a potential target for early prevention and intervention efforts.*

**What is Known:**

• *Short video use is increasingly common among children and may be associated with cognitive and emotional functioning.*

• *Maladaptive cognitive emotion regulation strategies, such as self-blame and catastrophizing, are linked to poorer emotional and behavioral outcomes in children.*

**What is New:**

• *Maladaptive cognitive emotion regulation strategies are associated with higher levels of short video addiction in children aged 10–14.*

• *Catastrophizing significantly predicts short video addiction, identifying a potential target for early prevention and intervention efforts.*

## Introduction

In recent years, short video platforms such as TikTok, YouTube Shorts, and Kwai have become integral components of children’s and adolescents’ digital environments, reshaping patterns of entertainment, communication, and daily media engagement [1]. Globally, the rapid expansion of short video platforms has been particularly pronounced among younger users. In China, for instance, the number of short video users surpassed 1.053 billion in 2023, representing 96.4% of all internet users, with 65.3% of minors actively engaging with short video content; notably, usage rates were highest among middle school students (67.9%) [1, 2]. These data underscore the early and pervasive exposure of school-aged children to short video platforms during a critical period of cognitive and emotional development.

Comparable trends have been reported in Türkiye, where internet penetration has reached 92% of households and social media use encompasses 73.8% of the population [3]. Empirical studies conducted with Turkish adolescents suggest that short video applications are widely used for entertainment, identity exploration, and peer interaction, often resulting in extended daily screen time [4–6]. While short video consumption is increasingly normalized in children’s everyday lives, growing concern has emerged about the potential psychological consequences of excessive, uncontrolled engagement, particularly during early adolescence.

Short video addiction (SVA) is conceptualized as a pattern of compulsive and repetitive short video consumption that interferes with academic functioning, emotional well-being, and social relationships [1, 7]. Accumulating evidence indicates that a substantial proportion of children and adolescents exhibit problematic levels of engagement. In a large-scale study involving 1492 middle school students, Ye et al. [2] reported that 21.3% of participants were classified as high risk for short video addiction, demonstrating impairments in attention regulation, academic concentration, and interpersonal functioning. These findings highlight that SVA extends beyond a benign leisure activity and may constitute a meaningful psychosocial risk factor in youth populations.

From a neurodevelopmental perspective, excessive exposure to short video content characterized by rapid pacing, salient visual stimuli, and frequent reward cues has been associated with heightened dopaminergic activity and increased limbic system engagement, particularly within the amygdala, alongside reduced activation of prefrontal regulatory regions [8, 9]. This imbalance may compromise inhibitory control and promote habitual media consumption, especially in children whose executive functions and self-regulatory capacities are still under development [10]. Neuroimaging and electrophysiological studies further suggest that repeated exposure to such content may disrupt attentional processes and emotion regulation mechanisms, with downstream effects on learning, sleep patterns, and social behavior [1, 11]. These neurocognitive vulnerabilities underscore the importance of examining psychological factors that may predispose children to problematic short video use.

Within this context, cognitive emotion regulation has emerged as a central construct in understanding children’s emotional adjustment and behavioral regulation. Cognitive emotion regulation strategies refer to the conscious cognitive processes individuals use to manage and interpret emotionally challenging experiences, encompassing both adaptive strategies and maladaptive strategies [12, 13]. A substantial body of literature indicates that adaptive strategies are associated with psychological resilience and academic functioning, whereas maladaptive strategies are linked to internalizing problems such as anxiety, depressive symptoms, and social withdrawal [14, 15]. Importantly, habitual reliance on maladaptive cognitive emotion regulation strategies may increase vulnerability to external coping behaviors, including excessive engagement with digital media.

Emerging empirical evidence suggests that cognitive emotion regulation strategies may play a predictive role in problematic short video use. Studies conducted with adolescents have demonstrated that individuals who predominantly employ maladaptive strategies, particularly catastrophizing and emotional suppression, report higher levels of short video addiction [16, 17]. Furthermore, these strategies have been shown to mediate the relationship between problematic short video use and depressive symptoms, suggesting that excessive short video engagement may function as an avoidance-oriented coping mechanism for managing negative emotional states rather than serving solely as a causal agent of emotional dysregulation [18, 19]. Such findings highlight the need to conceptualize short video addiction within a broader self-regulatory and emotion regulation framework.

Despite increasing scholarly attention to short video addiction, notable gaps remain in the literature. Most existing studies have focused on older adolescents or university students, limiting insights into younger age groups [20, 21]. Moreover, few studies have explicitly examined whether specific cognitive emotion regulation strategies predict short video addiction levels during early adolescence, a developmental stage characterized by heightened emotional reactivity, increasing autonomy, and ongoing maturation of executive control processes. Addressing this gap is critical for identifying early psychological risk factors and informing developmentally sensitive prevention efforts. Therefore, the present study aims to examine whether cognitive emotion regulation strategies predict short video addiction levels in children aged 10–14 and to explore the associations between specific strategies, particularly maladaptive patterns such as catastrophizing and self-blame, and short video addiction. By clarifying the direction and nature of these relationships, this study seeks to contribute to a more theoretically grounded and developmentally informed understanding of short video addiction in childhood.

### Research hypotheses

- **H**_**1**_**: **There is a significant relationship between short video addiction and cognitive emotion regulation strategies in children aged 10–14.
- **H**_**2**_**: **Cognitive emotion regulation strategies significantly predict short video addiction levels in children aged 10–14.

## Materials and methods

### Design

This descriptive and correlational study was carried out in accordance with the STROBE reporting guidelines [22].

### Participant

The study sample consisted of students aged 10–14 attending Sakarya/Akyazı Atatürk Middle School. The required sample size was estimated using G*Power software [23]. A moderate effect size was assumed for the relationship between the Demographic Information Form, SVAS, and CERQ-k. A point-biserial correlation model with a two-tailed test was used, with an effect size of |ρ|= 0.20, an alpha level of 0.05, and a statistical power of 0.95. Based on these parameters, the minimum sample size was calculated as 197 participants. The study was completed with 202 children. Participants were eligible if they were 10–14 years old, proficient in Turkish, provided parental consent, and voluntarily participated; those with hearing or visual impairments, incomplete data, or who withdrew at any stage were excluded from the study.

### Measures

The Information Form contained closed-ended questions regarding participants’ age, gender, grade level, number of siblings, presence of illness, daily smartphone, tablet, and computer use, parental education, and family income.

The Short Video Addiction Scale (SVAS) was originally developed by Ye et al. [2]. It assesses addiction-related behaviors and emotional responses associated with short video consumption of middle and high school students (10–18 years). The scale includes 10 items rated on a 5-point Likert scale, with higher scores reflecting greater addiction; no items are reverse-scored. The Turkish adaptation was conducted by Türk and Yıldırım [24], and the Cronbach alpha in the current study was 0.82.

The Cognitive Emotion Regulation Questionnaire–Child Form (CERQ-k), developed by Garnefski et al. [25] for middle school students aged 10–14 years, consists of 36 items assessing nine cognitive emotion regulation strategies: self-blame, acceptance, rumination, positive refocusing, refocus on planning, positive reappraisal, putting into perspective, catastrophizing, and blaming others. The Cronbach alpha of this study ranged from 0.64. t0 0.84. The Turkish adaptation was completed by Kurtoğlu Karataş and Güngör Aytar [26].

### Data collection process

Data collection was conducted between January and June 2025. Before initiating the study, the school principal was informed about the research and designated as the institutional liaison. In coordination with the school administration, sealed envelopes containing detailed study information and the Parent Informed Consent Form were distributed to families. Only students whose parents returned a signed consent form were deemed eligible. The principal assisted the research team in identifying class periods that would not interrupt academic routines, enabling data collection during appropriate times. After parental consent, children were verbally asked whether they wished to participate, and age-appropriate explanations were provided to ensure comprehension. Voluntary participation and the right to withdraw were clearly emphasized. Data were gathered through self-report questionnaires administered in the classroom over approximately 10–15 min. Students completed the measures.

### Data analysis

Data were analyzed using IBM SPSS Statistics 28.0 (IBM Corp., Armonk, NY, USA). Descriptive statistics, including frequencies, percentages, means, and standard deviations, were used to summarize demographic characteristics. The Kolmogorov–Smirnov test assessed normality, and subsequent parametric or non-parametric tests were selected based on variance homogeneity. Relationships between descriptive variables and scale scores were examined using appropriate statistical tests. Logistic regression analysis was conducted to determine the predictive value of SVAS and CERQ-k scores, with independent variables included only when VIF < 10, tolerance > 0.2, and condition index < 15. A *p*-value < 0.05 and *p* < 0.01 indicated statistical significance.

### Ethical issues

This study received approval from the Sakarya Applied Sciences University Ethics Committee (Approval No. E-26428519–050.99–152570) and was conducted in accordance with the Declaration of Helsinki. Parents were informed through sealed envelopes containing study details and a consent form, and only children with written parental consent were considered eligible. Verbal assent was then obtained from participating children after age-appropriate explanations were provided in the classroom. Participation was emphasized as voluntary, with the right to withdraw at any time. All data were anonymized prior to analysis, and the data owner authorized their use for research.

## Result

### Descriptive features of the children

Table 1 presents the descriptive characteristics of the participants. The mean age was 12.12 ± 1.24 years, and the average number of siblings was 2.58 ± 1.46. Daily smartphone use averaged 2.74 ± 2.45 h, while tablet and computer use averaged 0.74 ± 1.78 h and 0.58 ± 1.35 h, respectively. Most participants were female (52.97%) and in the 7th grade (30.20%). The majority reported no chronic illness (85.15%). The most common family income level was “income equals expenditure” (51.98%). Mothers (60.89%) and fathers (50.50%) most frequently had a primary school education (Table 1).

**Table 1:** The descriptive features of the children

Variable	*M* ± *SD*	Min–max
Age	12.12 ± 1.24	10.00–14.00
Number of the sibling	2.58 ± 1.46	0.00–7.00
Daily smartphone usage time *(hour)*	2.74 ± 2.45	0.00–12.00
Daily tablet usage time *(hour)*	0.74 ± 1.78	0.00–12.00
Daily computer usage time *(hour)*	0.58 ± 1.35	0.00–10.00
Gender	*n*	%
Female	95	47.03
Male	107	52.97
Class		
5th class	54	26.73
6th class	42	20.79
7th class	61	30.20
8th class	45	22.28
Having any diseases		
Yes	30	14.85
No	172	85.15
Family income		
Income less than expenditure	9	4.46
Income equals expenditure	105	51.98
Income less than expenditure	88	43.56
Mother education level		
Illiterate	8	3.96
Primary school	123	60.89
High school	58	28.71
University	13	6.44
Father education level		
Illiterate	6	2.97
Primary/middle school	102	50.50
High school	72	35.64
University	22	10.89

### Relationships between short video addiction and cognitive emotion regulation strategies

A significant positive correlation was identified between SVAS and Self-blame (*r* = 0.21, *p* < 0.05, 95% CI [0.08, 0.34]), indicating a small effect and suggesting that higher SVAS scores are associated with greater Self-blame. Similarly, SVAS was positively correlated with Putting into perspective (*r* = 0.20, *p* < 0.05, 95% CI [0.07, 0.33]), suggesting that this strategy increases as SVAS rises. A positive correlation also emerged between SVAS and Catastrophizing (*r* = 0.24, *p* < 0.01, 95% CI [0.11, 0.37]), indicating that Catastrophizing tends to increase with higher SVAS scores (Table 2).

**Table 2:** Pearson correlation results among SVAS and CERQ-k

Combination	*r*	95.00% CI	*n*	*p*
SVAS-Self-blame	.21	[.08, .34]	202	< .05
SVAS-Acceptance	.08	[− .06, .21]	202	.47
SVAS-Rumination	.12	[− .02, .26]	202	.24
SVAS-Positive refocusing	.15	[.01, .28]	202	.16
SVAS-Re-focus on the plan	.15	[.01, .28]	202	.16
SVAS-Positive reappraisal	.08	[-.06, .22]	202	.47
SVAS-Putting into perspective	.20	[.07, .33]	202	< .05
SVAS-Catastrophizing	.24	[.11, .37]	202	< .01
SVAS-Blaming others	.18	[.05, .31]	202	.08

### Predictive role of cognitive emotion regulation strategies on short video addiction

The linear regression model was significant, *F*(9,192) = 2.34, *p* = 0.016, R^2^ = 0.10, indicating that 10% of SVAS variance was explained by the nine CERQ-k subscales. Self-blame (*B* = 0.46, *p* = 0.05), Acceptance (*B* =  − 0.22, *p* = 0.35), Rumination (*B* = 0.04, *p* = 0.85), Positive refocusing (*B* = 0.22, *p* = 0.36), Re-focus on the plan (*B* = 0.004, *p* = 0.98), Positive reappraisal (B =  − 0.26, p = 0.27), Putting into perspective (*B* = 0.26, *p* = 0.34), and Blaming others (*B* = 0.03, *p* = 0.90) did not significantly predict SVAS. Catastrophizing significantly predicted SVAS (*B* = 0.59, *p* < 0.05), indicating that higher catastrophizing scores were associated with higher SVAS scores (Table 3).

**Table 3:** The predictive effects of cognitive emotion regulation strategies on short video addiction

Variable	***B***	***SE***	95.00% CI	***β***	***t***	***p***	Correlations			Collinearity statistics	
							Zero-order	Partial	Part	Tolerance	VIF
(Intercept)	11.62	3.21	[5.28, 17.96]	0.00	3.61	< .01					
Self-blame	0.46	0.24	[− 0.01, 0.94]	0.17	1.91	.05	.214	.137	.131	.611	1.637
Acceptance	− 0.22	0.23	[− 0.67, 0.24]	− 0.09	− 0.94	.35	.077	− .067	− .064	.539	1.855
Rumination	0.04	0.22	[− 0.39, 0.47]	0.02	0.19	.85	.123	.013	.013	.569	1.758
Positive refocusing	0.22	0.24	[− 0.26, 0.69]	0.08	0.90	.36	.150	.065	.062	.553	1.807
Re-focus on the plan	0.004	0.23	[− 0.45, 0.46]	0.002	0.02	.98	.148	.001	.001	.532	1.880
Positive reappraisal	− 0.26	0.24	[− 0.74, 0.21]	− 0.09	− 1.10	.27	.083	− .079	− .075	.697	1.435
Putting into perspective	0.26	0.28	[− 0.29, 0.81]	0.08	0.94	.34	.202	.068	.064	.591	1.692
Catastrophizing	0.59	0.28	[0.03, 1.14]	0.18	2.08	< .05	.241	.149	.143	.625	1.600
Blaming others	0.03	0.26	[− 0.49, 0.55]	0.01	0.12	.90	.183	.009	.008	.555	1.802

*F*(9,192) = 2.34; *p* = .016; *R*^2^ = .10, Durbin–Watson = 1.945

## Discussion

This study aimed to examine whether children’s cognitive emotion regulation strategies predict short video addiction among children aged 10–14. The findings demonstrated significant positive associations between short video addiction and maladaptive cognitive emotion regulation strategies, specifically self-blame, putting into perspective, and catastrophizing. However, consistent with the study hypothesis and regression model, catastrophizing emerged as the only cognitive emotion regulation strategy that significantly predicted short video addiction. This finding indicates that children who habitually engage in catastrophic thinking may be at greater risk of developing problematic short video use, rather than short video use directly shaping cognitive emotion regulation patterns. These results align with and extend existing literature suggesting that difficulties in emotion regulation constitute an important vulnerability factor for digital media–related addictive behaviors. Wang et al. [27] identified a significant association between short video addiction and emotional dysregulation in adolescents, emphasizing that impaired regulatory capacities may increase susceptibility to addictive media use. From this perspective, short video platforms may function as an easily accessible, short-term coping resource for children who struggle to manage negative affect, despite the potential for such use to exacerbate emotional difficulties over time. Yang, Ti, and Ye [16] reported that online social support seeking and emotional suppression significantly predicted short video addiction, suggesting that children and adolescents who rely on less adaptive regulatory strategies may turn to digital content for temporary emotional relief. The present findings further specify catastrophizing as a particularly salient cognitive risk factor, highlighting its role in amplifying perceived distress and promoting avoidant coping behaviors, such as excessive short-video consumption. Evidence from younger populations also supports this interpretation. Hussain, Ikram, and Aafreen [28] found that smartphone addiction in preschool-aged children was significantly associated with maladaptive cognitive regulation strategies, including catastrophizing and self-blame, indicating that the link between maladaptive emotion regulation and problematic technology use may emerge early in development. In contrast, the present study did not identify significant associations between short video addiction and adaptive strategies such as positive reappraisal, positive refocusing, or refocusing on the plan. This pattern suggests that children with higher levels of short video addiction may rely less on adaptive cognitive strategies, rather than actively using them to manage emotional distress. This interpretation is consistent with Guo et al. [29], who demonstrated that maladaptive strategies mediated the relationship between psychological distress and internet addiction, whereas adaptive strategies played a comparatively limited role.

Findings from the current study indicate that self-blame was positively associated with short video addiction, suggesting that children who tend to attribute negative experiences to themselves may be more vulnerable to problematic short video use. However, self-blame did not emerge as a significant predictor in the multivariate regression model, indicating that its explanatory power is limited when other cognitive emotion regulation strategies are considered simultaneously. This pattern underscores the multidimensional nature of short video addiction, which appears to be shaped by the combined influence of multiple cognitive and emotional processes rather than a single regulatory strategy. This finding is consistent with prior research suggesting that self-blame often functions as a correlational or contextual factor rather than a primary driver of digital media addictions. Liu et al. [30] reported a positive association between self-blame and social media addiction but emphasized that self-blame played a secondary role compared to other maladaptive strategies, such as externalizing blame. Zer et al. [31] found that although self-blame was associated with problematic social media use among university students, rumination and catastrophizing emerged as stronger predictors, highlighting their greater contribution to addictive patterns. Zsido et al. [32] demonstrated that individuals with elevated social anxiety were more likely to engage in self-blame alongside catastrophizing and rumination during social media use. They suggested that self-blame may reflect an internal emotional response accompanying digital engagement rather than a mechanism that directly motivates excessive use. These findings suggest that while self-blame may signal emotional vulnerability in children, it is unlikely to function as a dominant explanatory factor for short video addiction when compared with more cognitively pervasive strategies such as catastrophizing.

In the present study, putting into perspective was positively associated with short video addiction; however, this association did not reach statistical significance, indicating that this cognitive emotion regulation strategy does not independently predict short video addiction in children aged 10–14. This finding suggests that, although perspective-taking is generally conceptualized as an adaptive strategy, its protective role may be limited in the context of highly stimulating, immediately rewarding short-video platforms. Children’s efforts to cognitively downplay emotional experiences or compare them with less difficult situations may be insufficient to counterbalance the strong reinforcement mechanisms embedded in short-form digital content. This result is consistent with prior research demonstrating that adaptive emotion regulation strategies tend to show weaker associations with problematic digital media use compared to maladaptive strategies. Zer et al. [31], for example, reported that while self-blame and catastrophizing were significantly related to social media addiction, strategies such as putting things into perspective exhibited minimal explanatory power. Peker and Yıldız [33] found that suppression was a significant correlate of social media addiction, whereas more adaptive strategies, including cognitive reappraisal, showed weaker effects. Given that reappraisal shares conceptual similarities with putting into perspective, its influence on short video addiction may be indirect rather than direct. Rahimi et al. [34] demonstrated through structural equation modeling that the effects of adaptive cognitive emotion regulation strategies on social media addiction were primarily mediated by reductions in psychological distress. Accordingly, putting into perspective may function as a contextual or buffering strategy, exerting influence only under conditions of lower emotional strain or greater environmental support. These findings suggest that adaptive strategies alone may not be sufficient to predict short video addiction, reinforcing the importance of maladaptive strategies, particularly catastrophizing, as more salient predictors in this age group.

The findings of the present study indicate that catastrophizing emerged as the only cognitive emotion regulation strategy that significantly predicted short video addiction scores. Specifically, a one-unit increase in catastrophizing was associated with an average increase of 0.59 units in short video addiction, suggesting that children who habitually interpret negative experiences in exaggerated or worst-case terms are more vulnerable to problematic short video use. From a cognitive–emotional perspective, catastrophizing may heighten emotional distress and reduce perceived coping capacity, thereby increasing reliance on short-form digital content for temporary emotional regulation. This finding is consistent with existing literature linking catastrophizing to maladaptive digital media behaviors. Mihalca and Tarnavska [35] demonstrated that catastrophizing in adolescents was associated with heightened psychological distress and impaired social functioning, both of which may increase susceptibility to compensatory online behaviors. Zer et al. [31] reported a significant positive association between catastrophizing and social media addiction among university students, further noting its indirect impact through disrupted sleep patterns. Importantly, research focusing specifically on short video addiction has shown that catastrophic thinking styles may be reinforced by unmet psychological needs and emotional suppression, amplifying addictive tendencies toward highly stimulating digital content [16]. These findings suggest that catastrophizing may function as a key cognitive vulnerability factor underlying short video addiction, rather than a mere correlate. In the context of fast-paced, algorithm-driven platforms that offer immediate distraction and emotional relief, children prone to catastrophizing may be particularly inclined to engage in excessive consumption of short videos as a maladaptive coping strategy.

### Practical implications

This study demonstrates that short video addiction in children aged 10–14 is positively associated with maladaptive cognitive emotion regulation strategies, particularly self-blame and catastrophizing, with catastrophizing emerging as a significant predictor of short video addiction. These findings suggest that children who experience difficulties in managing negative emotions may be more prone to excessive engagement with short-form video content, potentially using such platforms as a maladaptive coping strategy. Similar associations between maladaptive emotion regulation and problematic digital media use have been reported by Yang et al. [16] and Wang et al. [27], supporting the notion that cognitive emotion regulation plays a critical role in children’s vulnerability to digital media–related problems.

From a practical perspective, the findings underscore the importance of incorporating emotion regulation training into preventive and intervention efforts. Programs that target maladaptive cognitive patterns, such as catastrophizing and self-blame, have been shown to enhance emotional resilience and psychological adjustment in school-aged children [12, 36]. Integrating these components into social–emotional learning (SEL) curricula may support the development of healthier coping strategies and reduce reliance on digital media for emotional regulation. In parallel, developmentally tailored digital literacy education is essential, as increasing children’s awareness of how short-form video algorithms shape attention and engagement may help mitigate excessive use [37].

Parental involvement also remains a critical component, given caregivers’ influence on children’s media behaviors. Supporting parents by guiding them on monitoring screen use, engaging in co-viewing practices, and establishing consistent, developmentally appropriate boundaries may further promote healthier media habits [38, 39]. At the school level, counseling services should be equipped to address digital media–related emotional difficulties, with professionals trained to identify early signs of problematic use and associated difficulties in emotion regulation. Cognitive–behavioral approaches targeting maladaptive strategies, such as catastrophizing and self-blame, may be particularly beneficial in this context [13, 14]. Finally, at the policy level, developing evidence-based guidelines on healthy digital media use, integrating SEL into national curricula, and promoting family-based interventions may collectively help reduce emotional risks and foster more adaptive digital engagement during this critical developmental period.

### Limitations and future directions

This study offers important insights into the relationship between short video addiction and cognitive emotion regulation strategies, though several limitations should be noted. The cross-sectional design precludes causal inference; future longitudinal research is needed to clarify directionality. The sample, drawn from a single school, may limit generalizability to different socio-economic or cultural groups. Additionally, unexamined factors such as parental digital habits, peer influence, and psychological well-being may also shape these relationships.

Future research should therefore adopt longitudinal designs, include broader and more diverse samples, and incorporate multi-informant assessments to strengthen validity. Examining additional variables such as parental media use, distress, and peer dynamics may offer a deeper understanding. Experimental or intervention studies evaluating digital literacy and emotion regulation programs are also recommended. Finally, neurocognitive investigations could further illuminate how short video use influences the development of self-regulatory processes in children.

## Conclusion

This study contributes to the growing literature on digital media use in childhood by clarifying the role of cognitive emotion regulation strategies in short video addiction among children aged 10–14. The findings demonstrate that maladaptive cognitive emotion regulation strategies, particularly self-blame and catastrophizing, are associated with higher levels of short video addiction. Among these strategies, catastrophizing emerged as a significant predictor of short video addiction, indicating that children who habitually engage in catastrophic thinking may be more vulnerable to problematic short video use. These results suggest that short video addiction should be understood not only as a media-related behavior but also as a manifestation of underlying self-regulatory and emotional coping processes. Given the widespread and early exposure of children to short-form video platforms, preventive efforts should prioritize strengthening adaptive cognitive emotion regulation skills alongside promoting healthy digital media habits. Educational and family-based interventions that support children in recognizing and managing negative emotions may reduce reliance on maladaptive coping strategies and, in turn, mitigate the risk of short video addiction. Future research employing longitudinal designs is warranted to further examine the temporal dynamics between cognitive emotion regulation strategies and short video addiction, as well as to explore potential developmental trajectories and long-term implications for children’s emotional well-being.

## Data Availability

No datasets were generated or analysed during the current study.

## Abbreviations

CERQ-k: Cognitive emotion regulation questionnaire – child version
SVA: Short video addiction
SVAS: Short video addiction scale
SEL: Social–emotional learning
STROBE: Strengthening the reporting of observational studies in epidemiology
VIF: Variance inflation factor
SD: Standard deviation
CI: Confidence interval
ANOVA: Analysis of variance
IBM SPSS: International Business Machines Statistical Package for the Social Sciences
